# The role of extracellular vesicles from different origin in the microenvironment of head and neck cancers

**DOI:** 10.1186/s12943-019-0985-3

**Published:** 2019-04-06

**Authors:** Changqing Xie, Ning Ji, Zhangui Tang, Jing Li, Qianming Chen

**Affiliations:** 1 Department of Oral and Maxillofacial Surgery, Xiangya Stomalogical Hospital & School of Stomatology, Central South University, Changsha, 410078 Hunan China; 2 State Key Laboratory of Oral Diseases & National Clinical Research Center for Oral Diseases & Chinese Academy of Medical Sciences Research Unit of Oral Carcinogenesis and Management & West China Hospital of Stomatology, Sichuan University, Chengdu, 610041 Sichuan China

**Keywords:** Tumor microenvironment, Extracellular vesicles, Exosomes Microvesicles, Cell-to-cell communication, Head and neck cancers

## Abstract

The proliferation and metastasis ability of tumors are mediate by the "mutual dialogue" between cells in the tumor microenvironment (TME). Extracellular vesicles (EVs), mainly exosomes and microvesicles, play an important role in achieving intercellular substance transport and information transfer in the TME. Initially considered "garbage dumpsters" and later referred to as "signal boxes", EVs carry "cargo" (proteins, lipids, or nucleic acids) that can redirect the function of a recipient cell. Currently, the molecular mechanisms and clinical applications of EVs in head and neck cancers (HNCs) are still at an early stage and need to be further investigate. In this review, we provide insight into the TME of HNCs, classifying and summarizing EVs derived from different cell types and illuminating their complex signaling networks involved in mediating tumor proliferation, invasion and metastasis, vascular angiogenesis and cancer drug resistance. In addition, we highlight the application of EVs in HNCs, underlining the special pathological and physiological environment of HNCs. The application of tumor heterogeneous EVs in saliva and circulating blood diagnostics will provide a new perspective for the early screening, real-time monitoring and prognostic risk assessment of HNCs. Given the concept of precise and individual therapy, nanostructured EVs are equipped with superior characteristics of biocompatibility, low immunogenicity, loadability and modification ability, making these molecules one of the new strategies for HNCs treatment.

## Introduction

Head and neck cancers (HNCs) is one of the most common malignant tumors in the world. The head and neck section addresses nonmelanoma skin cancer (NMSCs) of the head and neck as well as those malignancies that arise from the mucosal surfaces of the upper aero-digestive tract (UADT) and salivary glands, thyroid cancers are part of a separate section in the AJCC Cancer Staging Manual, eighth edition [1]. The HNCs described herein will mainly include almost all mucous malignancies of the oral cavity (e.g., tongue, buccal, gingiva, lip, and palate), oropharynx, larynx and nasopharynx [1]. In addition, the jaw, salivary glands, and maxillary sinuses are also included, and more than 90% HNCs are squamous cell carcinomas (SCCs) [2, 3]. Data from the International Agency for Research on Cancer (IARC), based on GLOBOCAN worldwide estimates, show that approximately 600,000 new cancer cases are reported (accounting for approximately 3% to 5% of all malignancies), and 350,000 cancer-related deaths occur (accounting for approximately 4% of the world total) worldwide each year, bringing serious challenges to social and public health [4–6].

Numerous epidemiological studies have reached a consensus that tobacco and alcohol consumption, both of which have synergistic effects, are the major risk factors for HNCs. In high-income countries, 71% and 37% of patients with oral cancer died from smoking and alcohol, respectively, compared with 33% and 14% in low- and middle-income countries [5, 7, 8]. A growing number of studies have suggested that betel-quid chewing with or without tobacco [9, 10], and human papilloma virus (HPV) infection (HPV16/18) are intimately related to the occurrence of HNCs. These two risk factors may lead to the younger age distribution and the increased rate of females in HNCs in recent years [11].

The treatment of local and early HNCs mainly involves resection with clear 1- to 2-cm margins [3]. In patients with advanced stage cancers and cervical lymph node metastasis, radical surgery combined with radiotherapy/chemotherapy adjunctive therapy is required [2, 3]. The strong invasion, migration and metastasis of HNCs leads to clinical treatment difficulty and poor prognosis. Over the past several decades, although there has been many innovations in HNCs treatment strategies, the overall 5-year survival rate is still only approximately 60% [2, 3, 5, 12]. Consequently, investigating the molecular mechanism and screening precise biomolecular markers of HNCs development is a tremendous challenge and opportunity.

Increasing numbers of studies have demonstrated that the interaction between tumor cells and the tumor microenvironment plays a crucial role in manipulating the tumor immune response, tumor progression and metastasis [13]. The tumor microenvironment has unique heterogeneity and diversity, which not only refers to the homeostatic intracellular environment of tumor cells but also the extracellular stromal cells (for example, fibroblasts, mesenchymal stem cells, various immune cells, vascular endothelial cells, etc.) and multiple tumor-promoting bioactive molecules [14]. Compared to the normal tissue environment, the tumor microenvironment has many different physical and chemical properties, which characterized by low oxygen, low pH and high interstitial pressure [15–18]. Due to this peculiarity, complex network signal communication between cells in the tumor microenvironment is triggered via several growth factors, cytokines, proteolytic enzymes and other active factors and is initially beneficial to tumor cell proliferation, migration, invasion, angiogenesis and radiotherapy/chemotherapy resistance. Among these biological mediators, extracellular vesicles (EVs), which function as information messengers, have gradually attracted the attention of researchers.

### Background of extracellular vesicles (EVs)

EVs are a subcellular structure of phospholipid bilayers membrane-enclosed vesicles. Numerous studies have shown that the cells of virtually all organisms (from prokaryotes to eukaryotes) can release the EVs to extra-environment in an autocrine or paracrine manner [19]. Initially considered "garbage dumpsters" and later referred to as "signal boxes", the entire history of the development of EVs can be divided into three periods. The first period was in the 1960s. Researchers extracted a precipitate from normal blood by high-speed centrifugation and confirmed that this precipitate, similar to the thromboplastic protein fraction, which is the predecessor of EVs, can reverse coagulation dysfunction [20]. Subsequently, it was further confirmed that activated platelets can release lipid-rich particles, referred to as “platelet dust”, which contain platelet factor 3 and can accelerate coagulation [21]. The second period was the 1970s and 1980s. Multiple studies have demonstrated the presence of membrane-like vesicle structures in other solid tissues, physiological fluids, and cell culture supernatants [22–24]. In addition to vesicles that originate directly from membrane shedding, researchers have revealed that vesicle structures are also derived from the intracellular secretion pathway of multivesicular endosomes (MVEs) or multivesicular bodies (MVBs), which are named exosomes [25, 26]. The third period is after the 1990s, during which the cell-cell communication function of EVs has gradually gained value, beginning with a landmark discovery of Epstein-Barr virus (EBV) infected B-cells capable of secreting molecules for the enrichment of major histocompatibility complex (MHC) class II-stimulated CD4+ T lymphocytes involved in the immune response [27, 28]. EVs are rich in lipids, polypeptides, proteins, RNA, DNA and other bioactive substances. EVs could be involved in various physiological and pathological processes, including cell cycle, apoptosis [29], angiogenesis [30], thrombosis formation [31], immune inflammation [32], fibrosis [33], and tumor development [34, 35].

The extraction methods for EVs have been very mature and commercialized [36]. Classical methods widely recognized by academic researchers, include ultracentrifugation [37], gel exclusion chromatography [38], immunoprecipitation [39], high performance liquid chromatography [40], flow cytometry [41], microfluidic chip [42] and other technologies, can acquire different subsets EVs. Western blotting, transmission electron microscopy, dynamic light scattering, and nanoparticle tracking analysis can identify these molecules. However, these methods cannot completely purify specific subpopulations, the products are often enriched with mixed vesicles in which one subgroup is dominant. These vesicles have more or less overlapping or similar particle sizes, morphologies, origins, contents, and functions. At present, the most classical EVs are mainly of two major types: exosomes and microvesicles [43].

### Biogenesis of exosomes

**Fig. 1:**
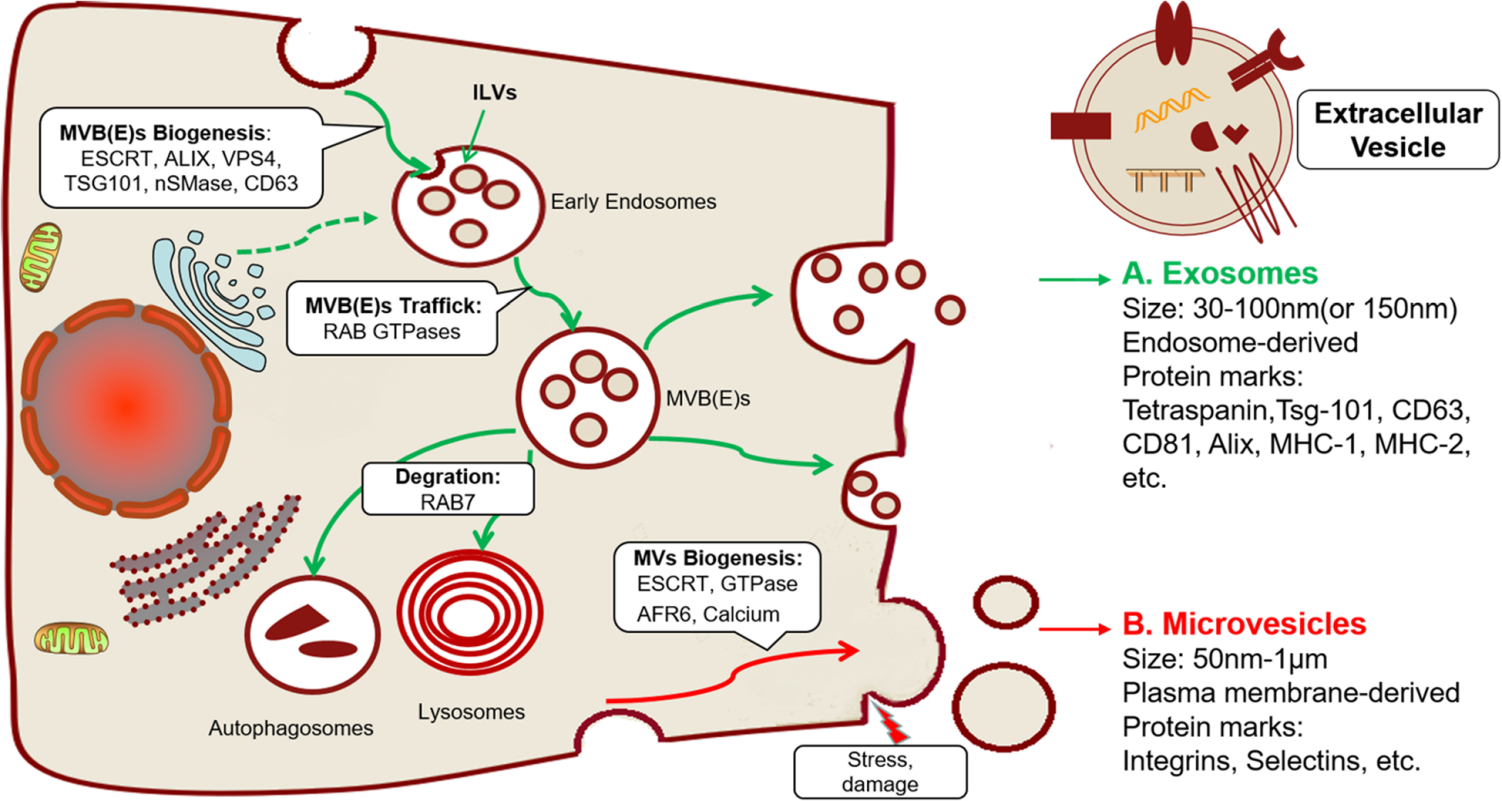
Biogenesis and characteristics of EVs. **a** Exosomes are endosome-derived vesicles that result from the dynamic equilibrium of membrane regeneration and degradation via the endosomal sorting complex required for transport (ESCRT) complex and its associated accessory proteins (ALIX, VPS4, and TSG101) or nSMase and CD63. Early endosomes inwardly regenerate to form intraluminal vesicles (ILVs) or exosome structures and then mature to multivesicular bodies (MVBs) or multivesicular endosomes (MVEs). RAB GTPases guide the intracellular binding and transport of MVB(E)s. Exosomes can be secreted by the fusion of MVB(E)s with cell membranes or be degraded in lysosomes and autophagosomes. **b** Microvesicles derive directly from the budding or shedding of the plasma membrane (PM) via ESCRT, AFR6 or external stress

Exosomes are endosome-derived vesicles with a particle diameter of 30-100 nm (or 150 nm) and are cup- or disc-shaped under electron microscopy [19, 27, 44]. The formation of endosomes is the result of the dynamic equilibrium of membrane regeneration and degradation [45] (Fig. 1a). The formation of early endosomes originating from membrane retraction accompanied the continuous inward generation and enrichment of intraluminal vesicles (ILVs), which could be consider as exosomes [28]. Early endosomes can evolve into MVEs or MVBs, which ultimately fuse with cell membranes to excrete intrinsic exosomes and other active substances or undergo degradation in lysosomes and autophagosomes [46, 47].

A variety of proteins and molecules are involved in the formation, content assembly and secretion of exosomes. The most explicit mechanism is the endosomal sorting complex required for transport (ESCRT) complex and its associated accessory proteins (ALIX, VPS4, and TSG101) [28, 48]. The general process is as follows. First, the ESCRT complex (0, I, and II) actively recognizes and sequester ubiquitinates proteins on the endosomal membrane and then inward regenerates the buds to form the ILV/exosome structure through membrane remodeling. Finally, ESCRT-III performs a cutting function to free the ILV/exosome [44]. Studies have also confirmed that the formation of exosomes can be independent of the ESCRT pathway. For example, tetraspanins CD63 [49] and lipid metabolism enzymes neutral sphingomyelinase (nSMase) [50] can also induce and assemble exosome contents.

The key protein that regulates the intracellular trafficking of exosomes is the RAB family proteins, which consists of more than 60 GTPases [28]. The diversity of individual RAB binding partner determines the specific vesicle transport route and creates the complexity of membrane trafficking [28, 51]. RAB5 and RAB21 are involved in early endosome transport and mediate endocytosis pathway while RAB7 regulates cargo trafficking from early endosome to late endosome and subsequently to lysosome for degradation [52]. RAB27 is consider as a pivot protein involving in tumor-associated vesicle trafficking and highly expressed in many tumors. In addition, a lot of RAB proteins are associated with exocytic pathway including RAB 3, RAB11, RAB26, RAB27, RAB37, RAB35 and RAB38 [19]. Another vesicle transport route is from trans-Golgi network (TGN) to the plasma membrane, which is mediate by RAB22 and RAB31. Different RAB subtype proteins can selectively regulate the transport of different exosomes and anchor MVBs on the cell membrane [53]. For example: RAB27 regulates the transport and release of exosomes from advanced endosomes rich in CD63, ALIX, TSG101 [54], whereas the release of early nuclear endosomes rich in Wnt, PLP, and TfR is associated with RAB11 and RAB35 [55]. Ultimately, MVBs fuse with the membrane for the release of exosomes into the extracellular environment.

### Biogenesis of microvesicles

Compared to exosomes, the biogenesis of microvesicles (ectosomes, microparticles) is relatively simple. These vesicles are formed directly from the budding or shedding of the plasma membrane (PM) and have particle diameters ranging from 50 nm to 10 μm [56]. The biogenesis of PM-derived vesicles are diverse and homologous to the exosomes (Fig. 1b). The crosslinking of membrane surface receptors induces membrane retraction through the ESCRT pathway to form early endosomes that differentiate into exosomes or sprouting to form PM-derived vesicles [28]. In addition, external stress (DNA damage and irradiation), changes in intracellular calcium levels [57], and GTPase ARF6 overexpression also can trigger the release of PM-derived EVs by remodeling the cytoskeleton [58].

### Dysregulation of EVs in the tumor microenvironment (TME)

Compared to the normal state,there is a difference between the population and contents of EVs in the pathological microenvironment [59]. When cells exposed to various stress responses (e.g. hypoxia, acidity, and nutritional deficiencies), the biogenesis, content sorting and release of the EVs could change, and these molecules become the active factors that initiate the signaling pathway. For example, GTPase RAB22A regulates the secretion of extracellular vesicles depending on the hypoxia-inducible factors (HIFs) in a hypoxic environment [60]. Additionally, fat production-related pathways could be activate, and this abnormal fat metabolism is conducive to the formation and release of cancer cell exosomes [61]. However, triglyceride accumulation in exosomes results in an "energy storehouse", which can influence epithelial remodeling and connectivity functions [62, 63], and therefore promote the proliferation and infiltration of cancer cells. Hypoxic-EVs contain various oncogenic molecules that participate in the blood vessel formation, cell metastasis and other signaling pathways that ultimately affect the development of cancer [62, 64, 65]. Moreover, under the stimulation of inflammatory factors, such as TNF-α, IL-1β, IFN-γ and LPS, endothelial cells can upregulate exosome secretion in a concentration-dependent manner [66]. Proteomic and gene expression analyses have shown that superoxide dismutase, VEGF, immune activation factor, and NF-kB signaling molecules were overexpress in EVs compared to normal endothelial cells [66, 67].

In summary, the mechanism of EVs produced by different cells and the characteristics of EVs produced under physiological and pathological conditions are different. Based on the specificity anatomy of the head and neck (e.g., the initial segment of the digestive tract and respiratory tract, the coexisting hard tissue and mucosal soft tissue, and the dynamic microenvironment of saliva and plaque microbes), EVs in the HNCs microenvironment have unique characteristics. Sequencing or proteomic analysis data could show the specificity results of HNCs-derived EVs (Table 1).

**Table 1:**
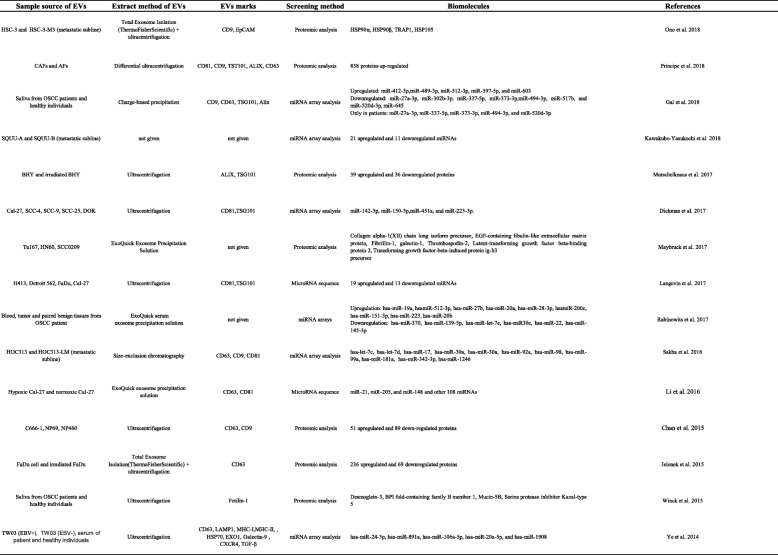
Sequencing or array and proteomic analysis datas results from different samples of HNCs-derived Evs

Oral Squamous Cell Carcinoma (OSCC) cell line: HSC-3, SQUU-A, Cal-27, SCC-4, SCC-9, SCC-25, H413, HOC313. Head and Neck cancers (HNCs) cell lines: SQUU, BHY,Tu167, HN60, SCC0209, Detroit 562, FaDu, TW03, C666-1. AFs: adjacent tissue fibroblasts. DOK: oral dysplastic cell line. NP69, NP460: immortalized nasopharyngeal epithelial cells

### Function of EVs in head and neck cancers

**Fig. 2:**
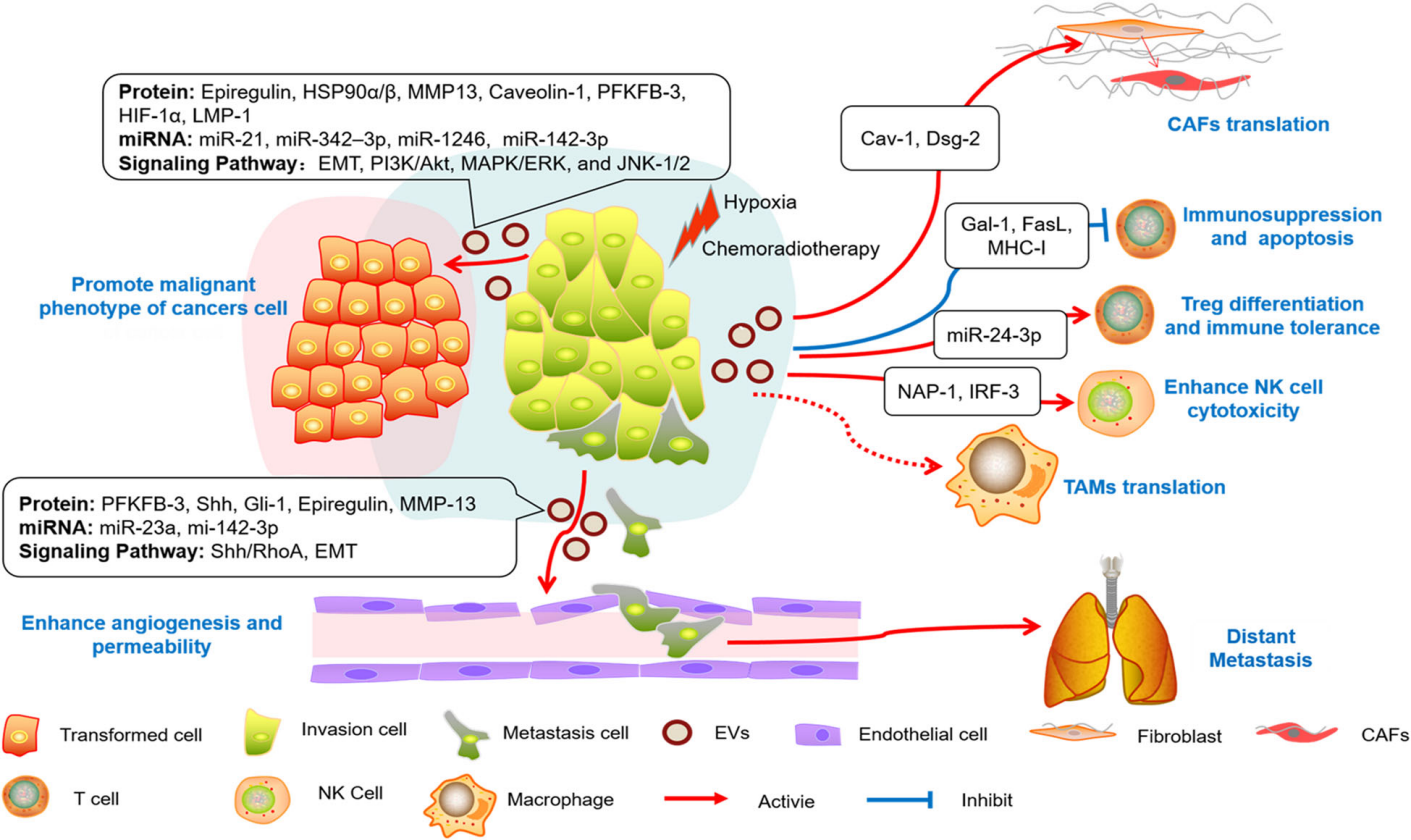
HNC-derived EVs in the cancer-to-stroma communication network. HNC-derived EVs can deliver various oncogenic proteins and noncoding RNA molecules to regulate surrounding cells. When the tumor cells are in hypoxic conditions or receive chemoradiotherapy, the gate of EV release can be opened, promoting the proliferation, migration and invasion, chemoradiotherapy resistance of cancer cells via EMT or other pathways. Endothelial cells actively absorb HNC-derived EVs, produce a variety of pro-angiogenic factors, induce angiogenesis, increase vascular permeability, and ultimately provide a venue for the distant metastasis of tumor cells. HNCs can package their "undesirable" substances (Cav-1 and Dsg-2) and assist the transformation of fibroblasts into CAFs. HNC-derived EVs can also regulate tumor immune responses. EVs can inhibit T-cell proliferation and Th1 and Th17 differentiation. Tregs are more susceptible to regulation by exosomal-miRNA-24-3p, which induces tumor immune tolerance. EVs activate T-cell surface receptors (FasL and MHC-I) to mediate the apoptosis of CD8+ T cells or via protein Gal-1-induced CD8+ T cells to display a suppressor phenotype. EVs induce the polarization of THP-1 to tumor-associated macrophages M2. Under certain conditions, exosomal NAP-1 could increase the cytotoxic activity of NK cells. HNC-derived EVs can mediate cancer-to-stroma communication to construct a premetastatic niche

Traditionally, tumor cells play a central leading role in the TME. However, in recent decades, studies have revealed that the successful proliferation and metastasis of cancer are inseparable from the "mutual dialogue" between tumor cells and stroma (Fig. 2 and Table 2).

**Table 2:**
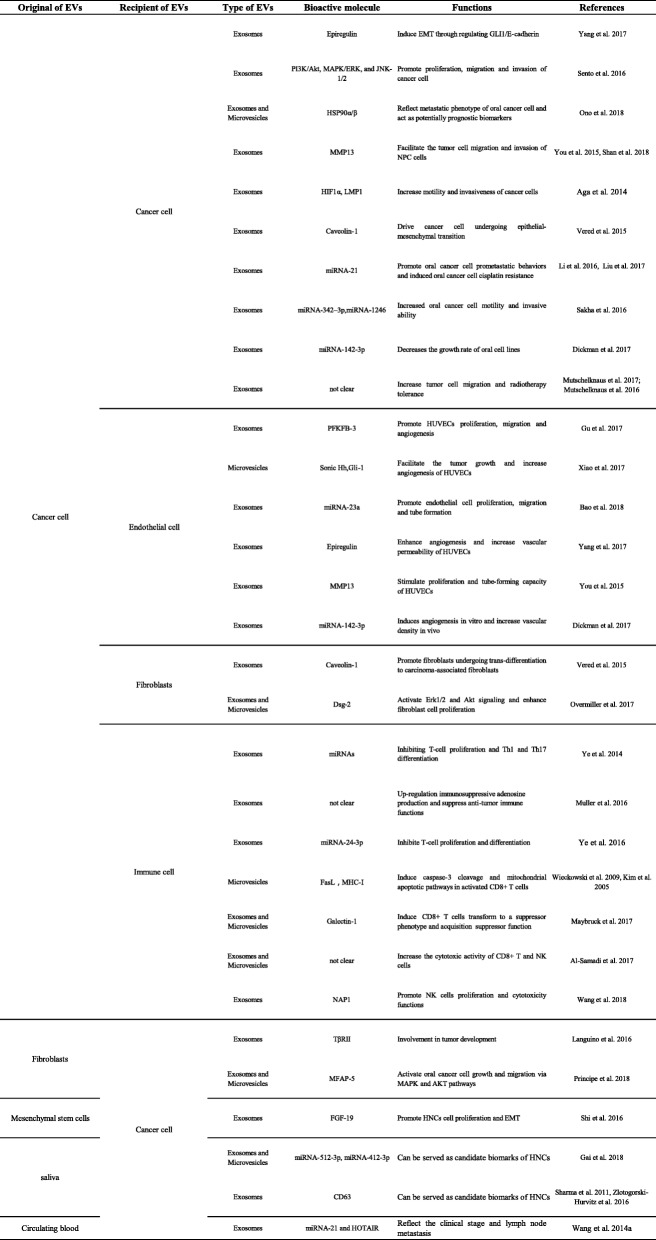
The function of different cell-derived EVs in the microenvironment of HNCs

### EVs in cancer-to-cancer communication

Heterogenous tumor cells can increase the motility and angiogenic activity of surrounding tumor cells by secreting EVs and creating a premetastatic microenvironment [59, 68], which is one of the key mechanisms of tumor recurrence and metastasis. By artificially mimicking the OSCC TME (arranging tumor cells in a three-dimensional myoma or collagen gel model), it was found that tumor-derived exosomes play an important role in regulating tumor epithelial morphology and function [69]. Exosomes could promote the formation of tumor epithelial islands and of the expression of interstitial cell characteristics, such as upregulating N-cadherin, downregulating E-cadherin and GLI-1, and eventually promoting the epithelial-mesenchymal transition (EMT) [70, 71]. Shinya Sento et al. demonstrated that OSCC-derived exosomes can self-uptake or become absorbed by surrounding tumor cells and then accelerate cell proliferation and invasion by activating AKT, MAPK/ERK, and JNK signaling pathways [72].

However, it is unclear what kind of active molecules in HNC-derived EVs play a role in the regulation of tumor progression. Proteomic analyses revealed that OSCC-derived EVs contain a series of tumor-associated proteins, including TRAP1, EGFR, HSP-90, and MMP-13. These proteins are significantly associated with the clinical stage and prognosis of OSCC patients and may serve as potential biomakers for metastatic phenotype and treatment of OSCC [73–75]. LMP1-positive exosomes and exosomal HIF-1α can also increase motility and invasiveness of HNCs via the EMT [76]. Andrew M. Overmiller et al. found that HNC-derived exosomes are rich in Dsg-2 [77]. The overexpression of Dsg-2 may promote tumor progression by degrading caveolins and matrix metalloproteinases could enhance EVs biogenesis and mitogenic effects [77–79].

Noncoding RNAs in HNCs-derived EVs are involved in the regulation of tumor progression [80, 81]. These noncoding RNAs may play an important function in promoting tumor development or inhibiting tumor progression. Hypoxia-induced OSCC exosomes can increase cancer cell migration and infiltration capacity. MiRNA-21 is significantly upregulated in hypoxic-derived OSCC exosomes, which is dependent on HIF-1α and HIF-2α pathways. MiRNA-21-enrich exosomes increase the expression of Snail and Vimentin proteins and downregulate E-cadherin levels in tumor cells, suggesting that OSCC can create a niche for distant transfer through the EMT [75, 82]. Highly metastatic and invasion oral cancer cells can transport exosome-derived miRNA-1246 and miRNA-200c-3p to the parental OSCC, which could target and bind to DENN/MADD Domain Containing 2D (DENND2D) and CHD9/WRN to promote tumor cell proliferation, metastasis, and invasion [83, 84]. More interestingly, the expression of miRNAs in EVs is a "selective" but not random process. OSCC cell lines can selectively package and discard miR-142-3p in exosomes. This process can activate the TGFBR1 pathway, which promotes endothelial cell angiogenesis and maintains the malignant states of tumor cells [85, 86].

Drug resistance is the main reason for the failure of chemotherapy in cancer patients. Interestingly, the exposure of head and neck tumor cells to some chemotherapy drugs (cisplatin, doxorubicin, and ROS-associated drugs) can lead to an imbalance of oncogenic contents in EVs and reduce the anti-proliferation and anti-metastatic effect of chemotherapy drugs [87, 88]. High chemoresistance OSCC cells can enhance drug resistance and reduce DNA damage by encapsulating miRNA-21 in exosomes and transporting it to target PTEN and PDCD4 [87, 89]. Radiation stress could increase the secretion of exosomes, and which could absorbed by unexposed tumor cells, hence increasing tumor cell migration and radiotherapy tolerance by provoking AKT-related signaling pathways or participating the repair of DNA double-strand break [90, 91].

### EVs in cancer-to-stroma communication

Tumor cell-derived extracellular vesicles, in addition to their uptake, can also transported to tumor stromal cells in a paracrine manner. Illumination of the mechanism of tumor-derived exosomes in mediating cell-cell communication could definitely provide a new perspective for the development of tumors.

### Cancer-to-endothelial cells

Folkman proposed the concept of "tumor growth depends on angiogenesis" as early as the 1970s [92]. Tumor angiogenesis activity has important value for histopathological grading, radiotherapy evaluation and prognosis. Therefore, patients undergoing surgery, radiotherapy and chemotherapy combined with tumor vascular blocking could effectively prevent the proliferation and metastasis of tumors. At present, studies on the mechanism of tumor angiogenesis have mainly focuses on a variety of pro-angiogenic factors (e.g., VEGF, FGF, PDGF) [93], but EVs also play an important role in the regulation of tumor angiogenesis [94].

The EVs derived from HNC cell can promote the malignant phenotype of tumor cells by delivering exosomal PFKFB3, Shh and other angiogenic proteins and activating the relevant model pathway to induce endothelial proliferation and tube formation [95, 96]. In addition, nasopharyngeal carcinoma (NPC) cell-derived exosomal miRNA-23a directly target the TSGA10 region to promote endothelial cell proliferation, migration and tube formation to regulate tumor growth [97]. Recent studies have shown that epiegulin-, MMP-13-, ICAM-1- or TSP-1-enriched exosomes could strengthen the release of vascular endothelial growth factors (VEGF-A, FGF-2, IL-8) and then downregulate junction-related proteins (claudins and ZO-1) that promote tumor angiogenesis and vascular permeability, becoming a potential passway system for distant metastasis of tumor cells [70, 71, 73, 98].

### Cancer-to-fibroblast cells

Cancer-associated fibroblasts (CAFs) are the major stromal cells in the TME [99]. Under physiological conditions, fibroblasts secrete a variety of factors (collagens, fibronectin, etc.) that play a role in maintaining the homeostasis of the extracellular matrix (ECM). In tumor microenvironment, fibroblasts cloud activated by tumor-associated chemokines (e.g., TGF-β, IL-6, and IL-8) and subsequently converted to CAFs. Tumor cell-derived exosomes can also assist in fibroblast translation to CAFs, resulting in a premetastatic microenvironment [100, 101]. Oral tumor cells can package their "undesirable" or "unhealthy" substances in EVs to relieve the damage caused by external environmental stimuli and simultaneously promote high expression of α-SMA and Twist by fibroblasts, suggesting that exosomes may mediate the EMT and regulate the conversion of fibroblasts into CAFs [79]. In order to adapt the hypoxia and hypo-nutrient conditions, tumor cells can achieve metabolic reprogramming by regulating the release EVs [102]. Hypoxia induces oral tumor cells or CAFs to secrete caveolin-1, trafficking by extracellular vesicles, which as a direct transcriptional target of HIF-1α and HIF-2α. On the one hand, EVs derived from tumor cells cause a crisis on oxidative stress of the TME. On the other hand, caveolin-1-null CAFs, induced by exocytosis or other pathways, provide multiple metabolic substrates (e.g., lactate, pyruvate, ketone) for tumor tissues. In brief, EVs may be involved in the construction of pseudo-hypoxic conditions of the TME and contribute to tumor development. Moreover, EVs from HNSCC encapsulate a large number of mitogenic proteins that regulate the proliferation of fibroblasts by various types of pathways such as EGFR, AKT, and ERK1/2, in a concentration-dependent manner [77].

### Cancer-to-immune cell

The effective amplification and metastasis of cancer cannot separated from the immune escape ability of cancer cell or treatment-mediated immune surveillance. Tumor cell-derived EVs are indispensable targets in the complex network of tumor immunity [103]. Tumor cell-derived EVs can suppress immune function, promote the differentiation of regulatory T cells and tumor-associated macrophages, and even replace tumor cells with immune cell attack to assist tumor cell immune tolerance and immune escape [104–106].

HNC-derived exosomes can prevent the proliferation of T lymphocytes and inhibit their differentiation into Th-1 and Th-17 cell subtypes and promote their conversion to Treg lymphocytes and myeloid-derived suppressor cells (MDSCs) [107]. Compared to other T-cell subtypes, Tregs are more susceptible to regulation by tumor-derived exosomes, leading to of the increased production of immunosuppressive adenosine [108]. HNC-derived exosomes enriched galectin-1 mediate the downregulation of STAT-1/-3 phosphorylation and upregulation of STAT-5 phosphorylation via the MAPK/ERK pathway or decrease expression of CD27/28-induced CD8+ T cells, displaying a suppressor phenotype [109]. Similar research proves that hypoxia inducing the upregulation of exosomal miRNA-24-3p in HNCs, which can repress FGF-11 to inhibit phosphorylation of the ERK and STAT proteins of T cell [110]. HNC-derived EVs function by endocytosis and activate T-cell surface receptors to regulate the transcription of immune-related genes. The surface proteins Fas-L and MHC-I of HNC-derived EVs mediate the apoptosis of CD8+ T lymphocytes [111–114]. The effects of oral tumor cell-derived exosomes on macrophages are uncertain. These vesicles can induce the polarization of THP-1 cell to tumor-associated macrophages M2 but have no significant effect on primary human macrophages [115]. Under certain conditions, EVs can also mediated antitumor immune responses. HNC-derived exosomes can upregulation NF-κB-activating NAP1 expression in NK cells, activate the expression and phosphorylation of IRF-3, and release multiple antitumor inflammatory factors (IFNs, CD40/80/86, and CXCLs) [116]. Overall, HNC-derived EVs mediate the bidirectional regulation of tumor immunity, suggesting that future immunotherapy may be based on the tumor microenvironment or individualized treatment of circulating EVs.

### EVs in stroma-to-cancer communication

**Fig. 3:**
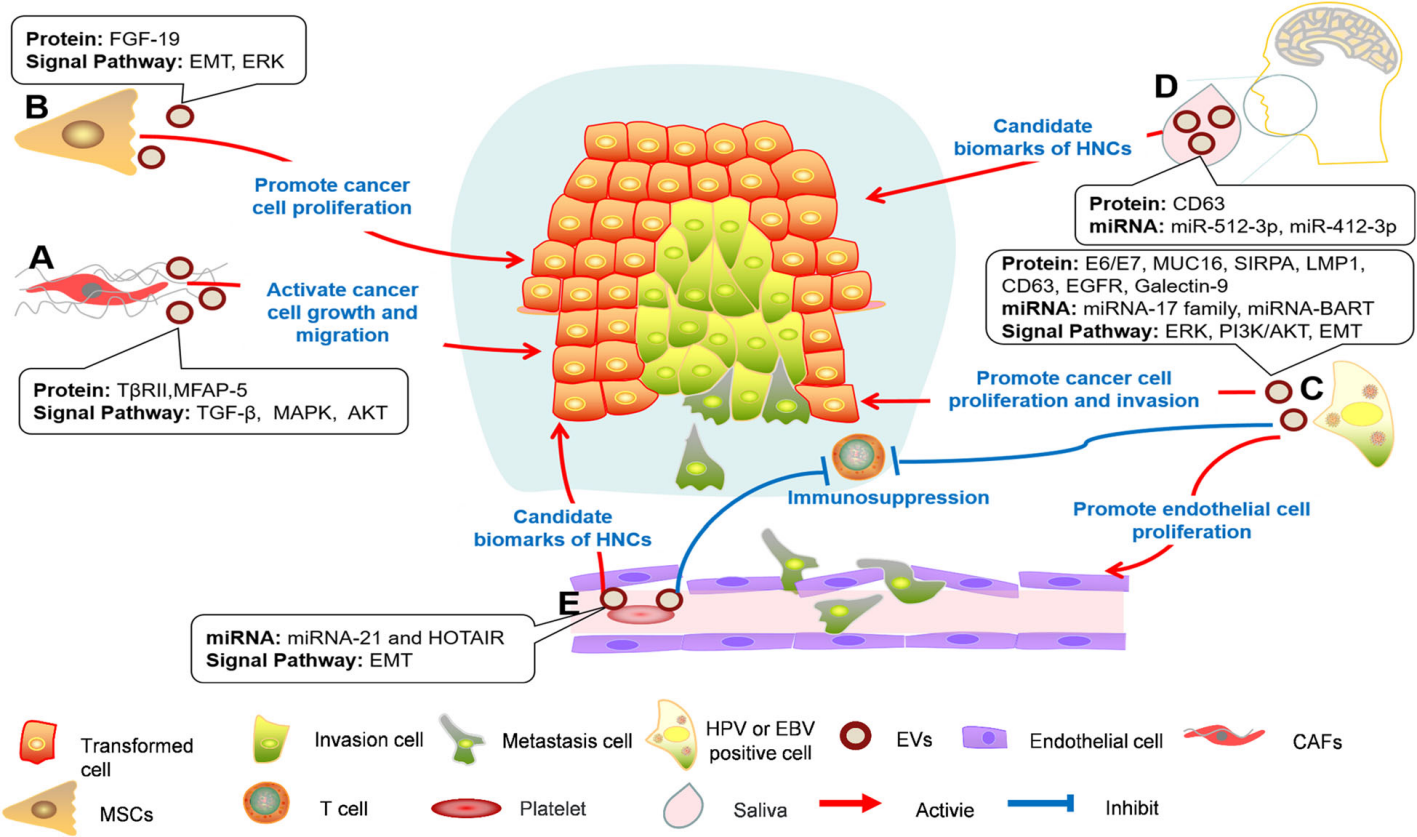
Different sources of EVs in the cancer-to-stroma communication network. Stromal cells are not bystanders in the development of tumors. **a** Cancer-associated fibroblast (CAF)-derived EVs loaded with TβRII and MFAP5, which reactivate the TGF-β signaling pathway, trigger the activation of MAPK and AKT signaling pathways and promote the proliferation and metastasis of OSCC. **b** Mesenchymal stem cell (MSC)-derived exosomal FGF-19 induces the EMT via the FGF-19/FGFR-4 signaling pathway and promotes tumor cell progression. **c** HPV and EBV can hijack cancer-derived EV production to regulate cell-to-cell communication and package onco-proteins or virus-coding miRNAs, such as E6/E7, MUC16, LMP1, EGFR, and miRNA-BART, which modulate the TME and promote tumor development. EVs from the saliva **d** and circulating blood **e** of patients with HNCs can be used not only as potential biomarkers to reflect the clinical stage of tumor pathology but also as carriers of active molecules involved in tumorigenesis

The transfer of EVs between tumor cells and stroma cells is not unidirectional but rather occurs through dynamic bidirectional and multidirectional complex signal network (**Fig.** **3**). Stromal cells are not a passive bystander in the development of tumors. Practically, stromal-derived EVs play a critical role in the reconstruction of ECM [59], dictate local and distant metastasis [117] and regulation of drug resistance in tumor cells [118].

TGFβ type II receptor (TβRII) in CAFs-derived exosomes can reactivate the TGF-β signaling pathway in OSCC cells, which is closely related to tumor heterogeneity and tumor progression [119]. The coculture of oral tumor cells with CAFs or their supernatants can drive the transformation of tumor cell cycle to S and G2-M phases, downregulate the expression of E-caderin, and promote the invasion and migration of OSCC cells through EMT [120]. Protein profiling revealed that the high expression of MFAP5, a protein component of extracellular microfibrils, in CAFs-derived exosomes triggers the activation of MAPK and AKT signaling pathways and promotes the proliferation and metastasis of OSCC cells [121] (Fig. 3a). In addition, FGF-19 is highly expressed in mesenchymal stem cell-derived exosomes (Fig. 3b), which can induce the EMT through activation of the NPC cell FGF-19/FGFR-4 signaling pathway and promote tumor cell progression [122].

### EVs derived from other sources

The head and neck are located in the beginning of the digestive and respiratory tract. Distinctive anatomical location makes the malignant tumor originating in this area to the pathogenic microbial environment. In recently decades, the ratio of HPV-positive oropharyngeal cancer (OPC) [3, 11] and EBV-infection NPC in HNC patients has increased (Fig. 3c).

Several studies have verified that HPV-positive cancer cells can dictate the expression levels of tumor genes and proteins in EVs to exert proliferative, anti-apoptotic and anti-senescent effects on surrounding cells [123, 124]. The persistent expression of E6/E7 protein in HPV-positive cancer cells can alter the miRNA pool in intracellular and exosomes; for example, the upregulated miRNA-17 family can inhibit P53/P21 expression levels and regulate tumor cell proliferation [125]. HPV-associated oropharyngeal cancer-derived exosomes packing MUC16, SIRPA and HPV-16-E7 proteins enhance the invasion of epithelial cells via the EMT [126].

EBV can hijack cancer-derived EVs production to regulate cell-to-cell communication and package viral components, such as LMP1 and CD63, which modulate the TME and promote tumor development [127, 128]. EVs released by EBV-positive NPC activate the ERK and PI3K/AKT proliferation pathway in cancer and endothelial cells via selective transport of LMP1, EGFR, virus-encoded miRNAs (BART miRNAs) [129, 130]. The C-terminal farnesylation of UCH-L1 is consider as a potential mechanism for EBV-positive NPC to release exosomes. Blocking the farnesylation of UCH-L1 can downregulate the release of LMP1-positive exosomes and inhibit tumor cell migration and colony formation [131]. In addition, EBV-positive exosomes promote galectin-9/tim-3 interactions and exert Th-1 suppressive functions [132].

### Potential application of EVs in head and neck cancers

#### **Application in the screening and diagnosis of** head and neck **cancers**

As a tool for liquid biopsy, EVs have unique advantages over circulating tumor DNA (ctDNA) and Circulating Tumor Cells (CTCs) [133, 134]. EVs highly reserve the original source of cellular biological information and easier to enrich and acquire [81]. More importantly, the protective effect of the double-layered membrane structure of EVs overcomes the problem of easy degradation of nucleic acids. In recent years, technology for search potential biomarkers of oral tumors in saliva EVs (Fig. 3d) and circulating EVs (Fig. 3e) has become increasingly mature [135]. Additionally, in the tumor microenvironment, either donor or recipient cells of EVs in the TME undoubtedly undergo genetic and epigenetic changes. Although primary tumor sample is standard starting material to perform genetic analyses, limiting factor is the obtained DNA cannot indicate genetic and epigenetic changes, such as acquired resistance to EGFR inhibitor, which is a major issue worldwide. Liquid biopsy is becoming a common alternative approach to perform molecular analyses, since it is less invasive than tumor biopsy and it is easily repeatable. cfDNA in EVs could be used to evaluate genetic and epigenetic changes in tumor before treatment but also to monitor clinical responses.

Compared with other solid tumors, in addition to substance exchange with circulating body fluids (blood, lymph), oral tumors also undergo saliva (including gingival crevicular fluid) erosion and biological effects. In oral tumor microenvironment, tumor-associated biomolecules can enter the saliva through blood circulation or directly through saliva components [136, 137]. Saliva components similar to blood, containing a large number of proteins and genetic material [138–141]. Previously, saliva was mainly diagnostic for HNCs (especially oral cancer), and an increasing number of experiments confirmed that saliva components can also be used to monitor and screen other tumors, such as pancreatic cancer [142], lung cancer [143], breast cancer [144]. The concentration of saliva-derived exosomes in oral cancer patients significantly increased compared to that in healthy populations and displays irregular morphologies and larger particle sizes [140, 145–147]. OSCC-derived saliva exosomes contain a variety of tumor-associated proteins involved in multiple signaling pathways, including tumor immune responses, cell proliferation, and metal transport [148]. Recent studies have shown that miRNA-512-3p and miRNA-412-3p were highly expressed in saliva exosomes of OSCC patients. Receiver operating characteristic curves show that these two miRNAs have a good discrimination power for OSCC diagnosis and may serve as potential candidate biomarker [149]. Oral cancer-derived salivary exosomes significant increase in the expression of CD63 and decrease in the expression of CD9 and CD81, which could serve as an indicator for cancer, even in the early stages of the disease [147]. Therefore, salivary fluid diagnosis has the virtues of convenient sampling, real-time performance, and noninvasiveness. Therefore, this technique has unique advantages in the early diagnosis of tumors, the detection of tumor progression and drug treatment evaluation [80, 150, 151].

The obvious upregulation of circulating EVs population in oral cancer patients positively correlated with IL-6 and TNF-α in tumor tissues. Tumor-derived microparticles can enhance the coagulation function of microparticles free plasma, which may promote venous thrombosis and involved in tumorigenesis [152]. High levels of miRNA-21 and lncRNA (HOTAIR) in serum-derived exosomes can reflect the clinical stage and lymph node metastasis of laryngeal squamous cell carcinoma, and evaluation of the expression of both molecules can increase diagnostic efficiency and accuracy [153]. A recent study showed that the plasma-derived exosomes in HNCs patients promote tumor cell proliferation, migration and invasion via the EMT. However, after photodynamic therapy, the patient plasma-derived exosomes can reverse this process and suppress malignant tumor cell characterization [154]. Plasma-derived exosomes from HNCs can also induce immune disorders to regulate the tumor microenvironment [155].

#### Application in prognostic risk evaluation

The prognostic evaluation of patients with metastatic tumors, including HNCs, is undoubtedly important for predicting tumor treatment outcomes, reducing recurrence and mortality, and prolonging survival. The inclusion of EVs as a prognostic factor for HNCs may become a trend of near future. Some EV-derived proteins (caveolin-1, HIF-1α, HSP-90, and MMP-2/9/13) shown closely related to the survival rate of patients with HNCs [74, 75, 79]. The level of extracellular vesicles in the serum may related to the immune function of patients, which could also indicate a poor prognosis for HNCs [112]. With the continuous updating and deepening of research, EV-associated DNA, protein, noncoding RNA, etc., expected to become useful for prognostic monitoring for HNCs, providing an effective basis for precise treatment.

#### Application in therapy strategy

As a natural intercellular information carrier, EVs have great application potential in the field of tumor therapy with its nanolevel molecular structure, unique host fingerprint, and properties of good biocompatibility [156]. Currently, the application of extracellular vesicles in clinical treatment can divided into the following areas. First, direct targeting of EV therapy prevents absorption by directly inhibiting the synthesis and secretion of tumor-associated EVs. However, such aggressive measures may produce side effects in patients, thus further investigations with larger clinical cohorts are required prior to EV therapy in the future. Second, EV-based immunization vaccines, involving the isolation and purification of autoimmune cell-derived EVs from tumor patients and antigen modification for subsequent return to the patient, can activate the ability of the immune system to kill tumor cells [157]. Third, an EV-based vector for cancer treatment drugs attributed to the low immunogenicity and stability of EVs [158]. Efficient and versatile EV anti-tumor delivery can circumvent the physiological barrier system to achieve the targeted treatment of drugs. Last, EV-based regenerative treatment is a research hotspot based on the use of EVs to solve the problem of stem cell survival and reduction in treatment [159].Some of these treatments have entered the clinical trial period. Unfortunately, there is no clear report on the use of EVs in the treatment of HNCs. We firmly believe that the application of EVs may be a solid supplement to the treatment of HNCs and should encourage further studies in this field.

## Conclusions

In summary, EV research is an emerging field that has been rapidly developing and has made some progress. However, the explicit molecular mechanism of EV biogenesis, content loading, intracellular and extracellular transport, and ultimately the regulation of disease progression has not yet explained. EVs are like a "double-edged sword" and have a close relationship with HNCs. On the one hand, EVs, as "alliances" of cancer, can promote the proliferation of cancer cells, assist the escape of cancer cells from the immune system or drug killing, and create a suitable microenvironment for the metastasis of cancer, which plays an important role in the development and progression of cancer. On the other hand, facilitating the occurrence of cancer exposes the existence of cancer and has become an effective means for the diagnosis and treatment of this disease. Currently, there is a debate about the definition of EVs and classification of subtypes and lack of standardization and unification of extraction techniques, which is a tricky issue for studies of EVs. With the advancement of precise and individual treatment, the use of EVs to establish the early screening and diagnosis of head and neck cancers enhance the evaluation of the prognosis and treatment effect, and promote the development of new anti-tumor drugs will be the aims of future exploration.

## Abbreviations

AJCC: American Joint Committee on Cancer
AKT: AKT serine/threonine kinase
ALIX: Apoptosis-linked gene 2-interacting protein X
ARF6: ADP ribosylation factor 6
CAFs: Cancer-associated fibroblasts
CHD9: Chromodomain helicase DNA binding protein 9
CTCs: Circulating tumor cells
ctDNA: Circulating tumor DNA
CXCLs: Chemokine (C-X-C motif) ligand
DENND2D: DENN/MADD Domain Containing 2D
Dsg-2: C-terminal fragment of desmoglein 2
EBV: Epstein-Barr virus
ECM: Extracellular matrix
EGFR: Epidermal growth factor receptor
EMT: Epithelial-mesenchymal transition
ERK: Extracellular regulated mitogen-activated protein kinase
ESCRT: Sorting complex required for transport
EVs: Extracellular vesicles
Fas-L: Fas ligand
FGF: Fibroblast growth factor
FGF-11: Fibroblast growth factor 11
FGF-19: Fibroblast growth factor 19
FGF-2: Fibroblast growth factor 2
FGF-4: Fibroblast growth factor 4
GLI1: GLI family zinc finger 1
HIF-1α: Hypoxia inducible factor 1 subunit alpha
HIF-2α: Hypoxia inducible factor 2 subunit alpha
HIFs: Hypoxia-inducible factors
HNCs: Head and neck cancers
HPV: Human papilloma virus
HSP-90: Heat shock protein 90
HSPs: Heat shock proteins
IARC: International Agency for Research on Cancer
ICAM-1: Intercellular adhesion molecule 1
IFNs: Interferons
IFN-γ: Interferon gamma
IL-1β: Interleukin 1 beta
IL-6: Interleukin-6
IL-8: Interleukin-8
ILVs: Intraluminal vesicles
IRF-3: Interferon regulatory factor 3
JNK: c-Jun N-terminal kinase
LMP1: Latent membrane protein
LPS: Lipopolysaccharides
MAPK: Mitogen-activated protein kinase
MDSCs: Myeloid-derived suppressor cells
MFAP5: Microfibril associated protein 5
MHC: Major histocompatibility complex
MMP-2/9/13: Matrix metallopeptidase 2/9/13
MMPs: Matrix metallopeptidases
MUC16: Mucin 16, cell surface associated
MVBs: Multivesicular bodies
MVEs: Multivesicular endosomes
NAP1: Kinase-associated protein 1; nSMase neutral sphingomyelinase
NF-kB: Nuclear factor kappa B
NPC: Nasopharyngeal carcinoma
OPC: Opharyngeal cancer
OSCC: Oral squamous cell carcinoma
PDCD4: Programmed cell death 4
PDGF: Fibroblast growth factor
PFKFB3: 6-phosphofructo-2-kinase/fructose-2,6-biphosphatase 3
PI3K: Phosphoinositide-3-kinase
PLP: Proteolipid protein
PM: Plasma membrane
PTEN: Phosphatase and tensin homolog
RAB: Ras-related protein
RAB11: Ras-related protein 11
RAB22A: Ras-related protein 22A
RAB27: Ras-related protein 27
RAB35: Ras-related protein 35
ROS: Reactive oxygen species
SCCs: Squamous cell carcinomas
Shh: Sonic hedgehog
SIRPA: Signal regulatory protein alpha
STAT: Signal transducer and activator of transcription
STAT-1: Signal transducer and activator of transcription 1
STAT-3: Signal transducer and activator of transcription 3
STAT-5: Signal transducer and activator of transcription 5
TfR: Transferrin receptor
TGFBR1: Transforming growth factor beta receptor 1
TGF-β: Transforming growth factor beta
TGN: Trans-Golgi neteok
TME: Tumor microenvironment
TNF-α: Tumor necrosis factor alpha
TRAP1: TNF receptor associated protein 1
TSG101: Tumor susceptibility 101
TSGA10: Testis specific 10
TSP-1: Tumor suppressor region 1
TβRII: Transforming growth factor beta receptor II
UCH-L1: Ubiquitin C-terminal hydrolase L1
VEGF: Vascular endothelial growth factor
VEGF-A: Vascular endothelial growth factor A
VPS4: Vacuolar protein sorting 4 homolog
WRN: Werner syndrome RecQ like helicase
ZO-1: Tight junction protein 1
α-SM: Actin, alpha 2, smooth muscle, aorta
